# Development of a Multidimensional Proteomic Approach to Detect Circulating Immune Complexes in Cattle Experimentally Infected With *Mycobacterium bovis*

**DOI:** 10.3389/fvets.2018.00141

**Published:** 2018-06-26

**Authors:** Syeda A. Hadi, Wade R. Waters, Mitchell Palmer, Konstantin P. Lyashchenko, Srinand Sreevatsan

**Affiliations:** ^1^Department of Pathobiology and Diagnostic Investigation, College of Veterinary Medicine, Michigan State University, East Lansing, MI, United States; ^2^National Animal Disease Center, Agricultural Research Service, United States Department of Agriculture, Ames, IA, United States; ^3^Chembio Diagnostic Systems Inc., Medford, NY, United States

**Keywords:** bovine tuberculosis, dual path platform, immune-complexes, mass-spectrometry, *Mycobacterium bovis*, mycobacteria, biomarkers, diagnostics

## Abstract

**Objective:** To evaluate a high-resolution method to identify pathogen-specific biomarkers in serum of calves infected with *Mycobacterium bovis*.

**Methods:** Serum samples from four calves infected with *M. bovis* were collected before and after infection at weeks 9, 14, 15, 31, and 36. Immune-complex-associated mycobacterial antigens in the serum were enriched using an immunochromatography method termed, dual path platform (DPP). All regions of antigen capture zones, that consisted of monospecific rabbit polyclonal antibodies raised against *M. tuberculosis* lysates, on DPP strips were excised and analyzed by multidimensional proteomics. The resulting proteins were then passed through 4 rigorous peptide quality filters-false-hits, decoys, non-*M. tuberculosis* complex proteins were all removed followed by individual quality check of those remaining. Peptides were then checked on NCBI's BLASTp for *M. tuberculosis* complex specificity.

**Results:** Proteins in 2 of the animals passed the multipronged-highly stringent peptide quality analysis. Animal#54 had 7 unique *M. tuberculosis* complex proteins at week 14 post-infection, while animal#56 had 4 at week 36 post-infection along with 1 immunoglobulin.

**Conclusion:** *M. tuberculosis* complex -specific peptides identified in this study were identified in 2 animals and at 2 separate time points post infection. Further studies with better enrichment protocols and using larger sample sizes and replications are required to develop a TB-specific diagnostic tool for bovine tuberculosis.

## Introduction

*Mycobacterium bovis* causes tuberculosis primarily in cattle but it is also zoonotic. Transmission to humans occurs through close contact with infected animals or via consumption of contaminated animal products (e.g., unpasteurized milk or dairy products) (1–3). The primary screening test used in the field is tuberculin-based skin test which is time-consuming, labor intensive and associated with low sensitivity and variable specificity. Variability in specificity is caused by species differences and technique being used (4, 5). Ultimately a false-positive can lead to a considerable financial burden on farmers deterring control measures. Thus, there is a need for highly specific rapid field tests that are cost effective.

Immune complexes are formed by the non-covalent binding of antigens with antibody molecules circulating real-time (6). Lyashchenko et al. (7), reported the presence of *Mycobacterium* specific immune complexes in cattle experimentally infected with *M. bovis* detectable by the dual-path platform (DPP) assay that utilizes polyclonal antibodies against *M. tuberculosis* whole-cell antigens. This provided an unprecedented opportunity to interrogate *M. tuberculosis* complex-specific antigens enriched by polyclonal tuberculosis-specific antibodies using high resolution technique of liquid chromatography followed by dual mass-spectrometry (LC-MS/MS). LC-MS/MS can detect proteins at abundances as low as 10^−15^ moles, thereby enabling discovery of circulating in infected animals. In the present study, high-resolution multidimensional mass spectrometry analysis of the DPP-captured immune complexes was evaluated for its ability to identify the captured *M. bovis*-specific peptides that may aid in the development of a highly accurate tuberculosis diagnostics for animals and humans.

## Materials and methods

Seven Holstein calves obtained from a TB-free herd in IA and housed in a biosafety level 3 (BSL-3) facility at the National Animal Disease Center, Ames, IA were infected at 11 months of age with 8 × 10^3^ CFU of virulent *M. bovis* (95-1315; USDA Animal Plant and Health Inspection Service [APHIS] designation) by aerosol. This strain of *M. bovis* was isolated from a white-tailed deer in Michigan, USA. Animals were sampled for serum at multiple time points pre- and post-infection over the next 11.5 months at which point they were euthanized (7, 8). Necropsy of all the calves revealed presence of gross lesions in multiple organs specific to bovine tuberculosis and bacterial culturing from infected tissues confirmed the presence of *M. bovis* in all 7 animals infected.

**Figure 1:**
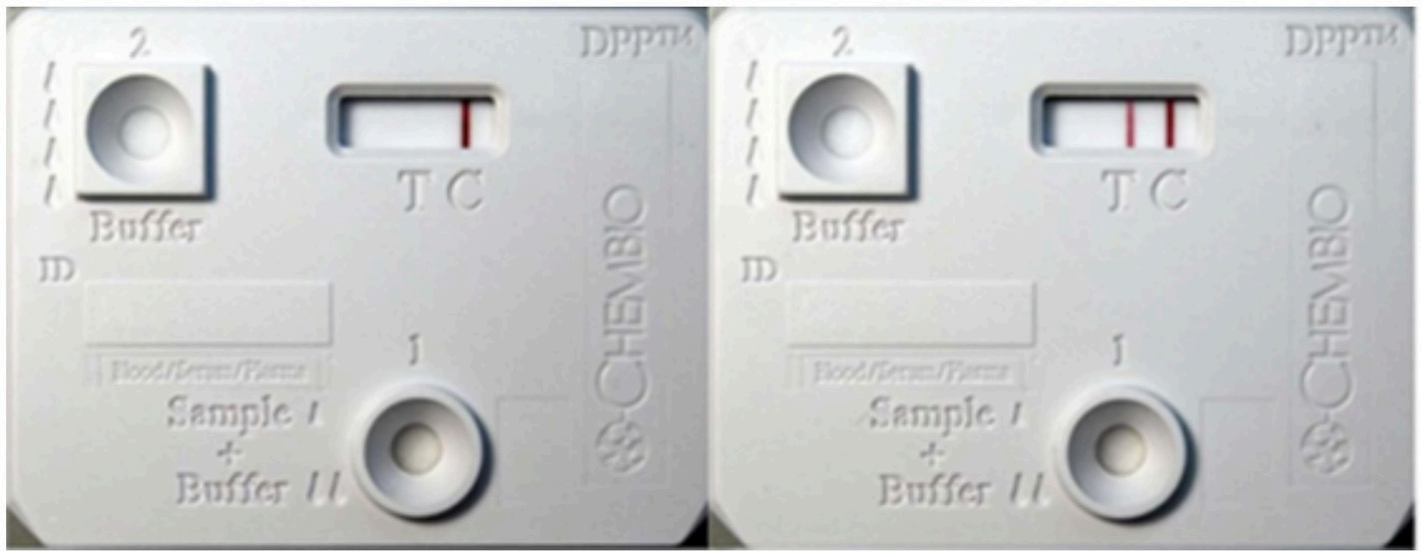
Dual-path platform assay kit showing positive and negative controls. Dual-path platform assay was used to detect circulating antigen-antibody complexes in calves infected with *Mycobacterium bovis*. The rabbit polyclonal antibodies immobilized on the test line(T) acted as the capture reagent for the circulating immune-complexes in the infected animal's serum as well as signal detector when coated onto nano-gold-particles. DPP strip case: **Left**: Negative Control (serum from uninfected animals), **Right**: Positive control.

In this pilot study we focused on 4 out of the 7 calves present in the original study (7), since they had the highest levels of circulating immune complexes to increase the probability of biomarker discovery. Pre- and post-inoculation samples collected at weeks 9, 14, 15, 31, and 36 were used to identify mycobacterial specific peptides. To characterize the circulating immune complexes-associated with *M. tuberculosis* complex, a rapid DPP-Ag assay was performed (Figure 1). The DPP antigen capture zone (test line) was coated with rabbit polyclonal antibodies raised against *M. tuberculosis* whole-cell lysate to enable capture of mycobacterial antigen-antibody complexes (7, 8). Pre-infection (baseline) sera from these four animals served as negative controls. Triplicates of each time points from every animal were made pre and post-infection (which summed up to 27 DPP-Ag assay strips for analysis) for each week 0, 9, 14, 31 and 36. A 50 μL aliquot of serum sample was placed on three independent DPP-Ag strips for each time-point, to allow for antigen enrichment of molecules on the capture zone, which were then processed as one single sample to allow for maximum enrichment, enhanced sensitivity, efficient use of the LC-MS/MS and improved proteomics profile generation.

The immune-complex capture zone of 2 mm width was excised and analyzed by LC-MS/MS analysis performed at University of Minnesota's Center for Mass Spectrometry and Proteomics (CMSP). Triplicates of DPP-assays for every animal were pooled for analysis. A region 2 mm upstream of the antigen capture zone (or the DPP test line) was also analyzed by LC-MS/MS. The enormous volume of peptide data generated by LC-MS/MS was passed through a series of stringent filters before the final candidates were considered.

First, PEAKS (Bioinformatics Solutions Inc.) software was used to query peptides generated in each triplicate-pooled-sample through LC/MS-MS against a database that included all documented peptides from *M. tuberculosis* Complex, cattle and rabbit proteins. These results were then analyzed by Scaffold (version Scaffold_4.7.5, Proteome Software Inc., Portland, OR) to validate all MS/MS based peptide identifications and to allow combined visualization of all sample results. All identified peptides were compared against a decoy database (generated in Scaffold_4.7.5), consisting of randomized peptide sequences, to remove any spurious hits. Second, any protein that matched against the decoy database, was removed from further analysis. We focused only on the *M. tuberculosis* complex proteins because they offer highest possible specificity for bovine tuberculosis diagnostics. The third filter was based on an individual quality check of the proteins with in Scaffold. Peptide identifications were accepted in Scaffold if they could be established at greater than 95.0% probability by the Peptide Prophet algorithm (9) with Scaffold delta-mass correction. Protein identifications were accepted if they could be established at greater than 95.0% probability and contained at least 2 identified peptides. Proteins that contained similar peptides and could not be differentiated based on MS/MS analysis alone were grouped to satisfy the principles of parsimony. Peptides and proteins that were selected in the third filter had percent probabilities varying from 74 to 100%. The fourth and the last filter was the identification of *M. tuberculosis* complex specificity using the National Center for Biotechnology Information (NCBI)'s non-redundant database BLASTp (basic local alignment search tool for proteins) analysis where two aspects were investigated: (1) *E*-value (<1e^−10^) and (2) the species match of the peptides. If the proteins matched with any other bacteria other than *M. tuberculosis* complex, they were excluded from further consideration. Additionally, if any peptides had an *E*-value higher than 1e^−10^, which suggested that the species match was likely non-random, they were also removed from further consideration. This last filter was excessively stringent as it eliminated most of peptide hits discovered after decoy database search. Some of the peptides eliminated may still be useful in a future validation study.

The same pipeline was followed for identifying cattle specific immunoglobulins, where immunoglobulins were passed through all the filters described for *M. tuberculosis* complex proteins. Additionally, the proteins that overlapped between pre-infection and post-infection test-lines were excluded as it suggested that they were not associated with the *M. bovis* infection, rather existed in the background.

## Results and discussion

The peptides generated from LC-MS/MS analysis resulted in identification of 26,945 proteins. Forty-nine percent of these were eliminated after the decoy database search. Of these, 3.73% were identified with the *M. tuberculosis* complex repertoire, 26.02% proteins were of host (bovine) origin and 21.35% were of leporine origin. DPP strips of all post-infection samples, except at week 31, had *M. bovis* proteins. After analysis, 11 *M. tuberculosis* complex-specific proteins were identified in two *M. bovis*-infected animals (Table 1). At week 14 (post-infection) serum from animal #54 showed 7 proteins that corresponded to peptides in *M. tuberculosis* complex with a BLAST *E*-value lower than 1e^−10^ (Table 2). At week 36 post-infection, serum of animal #56 had 4 proteins that corresponded to *M. tuberculosis* complex with an E-value lower than 1e^−10^(Table 2).

**Table 1:** Enumeration of pathogen-derived proteins detected by mass spectrometry from DPP-Ag assay strips processed with serum samples from cattle experimentally infected with *Mycobacterium bovis*.

**Animal ID**	**Pre-infection**			***Mycobacterium bovis* infection**			
	**DPPAg result^a^)**	***Mycobacterium bovis* proteins identified^b^**	**Cattle specific immunoglobulins**	**Week post-inoculation**	**DPP-Ag result^a^**	***Mycobacterium bovis* proteins identified**	**Cattle specific immunoglobulins**
51	0	0	0	9	124	0	0
54	0	0	0	14	788	7	0
				15	772	0	0
56	0	0	0	36	485	4	1
57	0	0	0	31	447	0	0

a *DPP reader data (reflectance) in relative light units obtained as described (7)*.

b *Pooled DPP-Ag strips processed with pre-infection sera from four calves*.

**Table 2:** List of Mycobacterium tuberculosis complex-specific high confidence proteins at week 14 and week 36 and cattle immunoglobulin at week 36 that passed exclusion criteria.

***Mycobacterium tuberculosis* complex proteins at wk14**	**Peptide sequences used for BLASTp analysis**
Acyltransferase	QDGSASYDAAVR-MLKAGELVGVYPEATISR
Esterase	VFGAADPR-FACVVRAFASMFPGR
LLM class F420-dependent oxidoreductase	QKDYDEYGYR-FGTAGSRLDDLAAPLPR
Transposase, partial	MDPTEDQARALAR-VTGIGTVKPSLRVLR
Transcriptional regulatory protein embr2	FGILGPLEISAGFRSLPLGTPK-SPLGRLPLR
Hypothetical protein Mb3478	GASPATAAR-LPPALNPDDADALPTTDRLTTR
Polyketide synthase	DGDRVLAIVR-LVDAPLPSWTHRTLMLSR-MFNSLGIQYGPAFSGLVAVHTAR-LFVVTRSAASVLPSDLANLEQAGMR
***Mycobacterium tuberculosis*** **complex proteins at wk36**	**Peptide sequences used for BLASTp analysis**
Helicase helz	VYAHHGGARLHGEALRDHLER-RGNVLAAMAKLK-IDEMIEEKKALADLVVTDGEGWLTELST
Hypothetical protein Mb1791	FGVTINDVVVALCAGALRR-VPSQISDPAQR
Hypothetical protein Mb2390c	HGHGRDVAAHR-TGGHRQASSRIK-HQKPGDVPRDPRC
Chromosome partition protein Smc	LDTMAANLARLTDLTTELR-LAVRTAEER
**Cattle Immunoglobulin at week 36**	**Peptide sequences used for BLASTp analysis**
PREDICTED: killer cell immunoglobulin-like receptor, three domains, long cytoplasmic tail, 2 isoform X2	GEMLTSGHAPADFVIGPMTLASAGTYR

*The proteins that did not comply with exclusion criteria were removed such as non-Mycobacterium tuberculosis complex proteins, decoy proteins, low quality proteins and proteins that did not identify with Mycobacterium tuberculosis complex when NCBI's non-redundant database BLASTp (basic local alignment search tool for proteins) analysis was used. The same pipeline was used to identify cattle immunoglobulins. The hyphen represents the space given in NCBI's BLASTp search that allowed to account for the presence of other amino acids in between the peptides that formed the proteins*.

At week 14 post-infection in animal #54 polyketide synthase was detected, which plays a role in the growth of the bacteria and is considered a potential virulence factor (10). The detection of this protein at such an early stage in *M. bovis* infection agrees with other studies (11, 12) where polyketide synthase was detected through different techniques but at similar time points. Lamont et al. (11), showed that polyketide synthase can be used as a useful marker for detecting *M. bovis* infection in a multi-cut off fashion, based on the prevalence of the disease.

Killer cell immunoglobulin-like receptor (1e^−18^) in animal #56 at week 36 corresponded to cattle (*Bos taurus*) specific immunoglobulin, alone passed all analysis filters. Even though pre-infection DPP-assays from all 4 animals were pooled together to enhance the probability of capturing all mycobacterial circulating immune complexes at base-line to compare them with proteins detected post-infection, no immune-complexes were detected at base-line.

The panel of mycobacterial proteins and cattle specific immunoglobulin reported in the present study may be specific to the infection stage at which they were detected, as the proteins seen at week 14 did not overlap with those detected at week 36 post infection. Alternatively, since these distinct proteins sets were found in two different animals, they could be a result of animal-to-animal variation in host response to the infection.

A major limitation of our study was sample size. Since the LC/MS-MS analysis itself was expensive and limited amounts of infected animal sera were available, multiple replications on the same animals were not possible. Additionally, multiple logistical and financial issues precluded us working with larger sample sizes: (1) working with agricultural animals for experimental infection with a BSL-3 pathogen and (2) Expenses associated with a BSL-3 cost, animal costs as well as personnel. Thus, to compensate for this limitation triplication of every animal's sample was performed.

Furthermore, the use of an antiserum derived against *M. tuberculosis* may have compromised specificity of our approach to detect *M. bovis* specific antigens, although these organisms are genetically very closely related. Future analysis though should include multiple replications of experimental infections followed by DPP assay and LC-MS/MS to discover *M. bovis* specific peptides in a reproducible and accurate fashion. Furthermore, a field validation on multiple exposure levels in outbreaks would be necessary for this technology to be applicable in routine bovine TB diagnostics.

In conclusion, the panel of 11 proteins reported in this study are specific to *M. bovis*. Further studies with more robust enrichment methods and larger sample sizes would be required to confirm these findings. Further validation of the identified circulating immune-complexes in naturally infected cattle would enable us to effectively and broadly apply the DPP technology in field.

## Ethics statement

All studies were approved by the National Animal Disease Center Animal Care and Use and Institutional Biosafety committees and performed under appropriate project licenses within the conditions of the Animal Welfare Act.

## Author contribution

WW and MP conducted the animal infection and testing studies. KL developed the DPP lateral flow devices. SS and SH developed protocols for extraction of total proteins from DPP devices, performed proteomics and data analysis. WW, SH, KL, and SS wrote the paper.

### Conflict of interest statement

KL is employed by Chembio Diagnostic Systems, Inc. The remaining authors declare that the research was conducted in the absence of any commercial or financial relationships that could be construed as a potential conflict of interest.
